# Has the Phase of the Menstrual Cycle Been Considered in Studies Investigating Pressure Pain Sensitivity in Migraine and Tension-Type Headache: A Scoping Review

**DOI:** 10.3390/brainsci11091251

**Published:** 2021-09-21

**Authors:** Francisca Curiel-Montero, Francisco Alburquerque-Sendín, César Fernández-de-las-Peñas, Daiana P. Rodrigues-de-Souza

**Affiliations:** 1 Department of Nursing, Pharmacology and Physical Therapy, Universidad de Córdoba, 14004 Córdoba, Spain; n82cumof@uco.es (F.C.-M.); falburquerque@uco.es (F.A.-S.); drodrigues@uco.es (D.P.R.-d.-S.); 2 Maimonides Biomedical Research Institute of Cordoba (IMIBIC), 14004 Córdoba, Spain; 3 Department of Physical Therapy, Occupational Therapy, Rehabilitation and Physical Medicine, Universidad Rey Juan Carlos, Alcorcón, 28922 Madrid, Spain; 4 Cátedra Institucional en Docencia, Clínica e Investigación en Fisioterapia: Terapia Manual, Punción Seca y Ejercicio Terapéutico, Universidad Rey Juan Carlos, 28922 Alcorcón, Spain

**Keywords:** tension-type headache, migraine, pain sensitivity, women, menstrual cycle, hormones

## Abstract

Objective: The aim of this scoping review was to identify if the phase of the menstrual cycle was considered in observational studies comparing pressure pain sensitivity between women with migraine or tension-type headache (TTH) and headache-free women. Methods: A systematic electronic literature search in PubMed, Medline, Web of Science, Scopus, and CINAHL databases was conducted. Observational studies including one or more groups with TTH and/or migraine comparing pressure pain thresholds (PPTs) were included. The methodological quality (risk of bias) was assessed with the Newcastle-Ottawa Scale. Authors, objectives, inclusion/exclusion criteria, size sample, female sample, tool to assess PPTs, mean age, and the use of any medication were extracted and analyzed independently by two authors. Results: From a total of 1404 and 1832 identified articles for TTH and migraine, 30 and 18 studies satisfied the criteria and were included. Nineteen (63.4%) studies assessing TTH patients and eleven (61.1%) assessing migraine patients showed a high risk of bias. The most common flaws were attributed to improper selection of control and control over other additional factors. Based on the systematic review, just one study including TTH and one including migraine patients considered the menstrual cycle. Conclusion: The results of this scoping review identified that the phase of the menstrual cycle has been rarely considered in studies investigating sensitivity to pressure pain in primary headaches, such as TTH or migraine, although there is evidence showing the relevance of the phase of the menstrual cycle in pain perception.

## 1. Introduction

Headache is a condition ranked among the top ten causes of disability-adjusted-life-years (DALYs) [1]. Migraine and tension-type headache are probably the most prevalent primary headaches. Tension-type headache (TTH) is the second most prevalent disorder worldwide [2]. Similarly, migraine has been ranked the third-highest cause of disability worldwide in both genders under the age of 50 years [3]. Both headaches feature the presence of pressure pain hyperalgesia [4,5]. This assumption is based on the results from several studies investigating sensitivity to pressure pain in both TTH [5,6,7,8,9,10,11] and migraine [12,13,14,15]. Additionally, migraine is also characterized by an increased sensitivity to visual or auditory stimulus during but also outside of the headache attack [4,16].

It is suggested that the menstrual cycle influences pain sensitivity [17]. During the luteal phase, the estrogen levels decrease and the levels of progesterone increase. In the luteal phase, pain thresholds are higher than in the follicular phase and during the menstruation [18]. In addition, pain inhibition is also higher during the ovulatory phase as compared to the menstrual and luteal phases [19]. Higher concentrations of progesterone decrease sensory neurotransmission since GABAergic tone is higher during the luteal phase and declines prior to the menstrual bleeding [20,21]. The role of the GABAergic system involves reducing the neuronal excitability of the nervous system [20,21]. Therefore, since the menstrual cycle affects pain sensitivity, studies investigating pressure hyperalgesia in primary headaches, such as migraine and TTH, should consider this “status”. For instance, studies comparing pain sensitivity between women with TTH or migraine and headache-free women should assess all participating women in the same phase of the cycle (e.g., menstruation-bleeding, luteal phase, ovulation, or follicular phase) to decrease the influence of the menstrual cycle on pain sensitivity between the comparisons. This is highly relevant since both TTH and migraine are more prevalent in women and gender differences are commonly seen [22,23].

No review has previously investigated if studies investigating pressure pain sensitivity in women with migraine or TTH have systematically considered the menstrual cycle. Therefore, the objective of this scoping review was to identify if the phase of the menstrual cycle was considered in observational studies comparing pressure pain sensitivity between women with migraine or TTH and headache-free women.

## 2. Materials and Methods

This scoping review was conducted following The Preferred Reporting Items for Systematic Reviews and Meta-Analyses Extension for Scoping Reviews (PRISMA-ScR) [24]. It was prospectively registered on the Open Science Framework Registry with the identifier DOI 10.17605/OSF.IO/SAFPQ and the link https://osf.io/r4uak (accessed on 18 December 2020).

### 2.1. Identify the Research Question

The main research question of the current scoping review was: Is the menstrual cycle taken into consideration in observational studies comparing pressure pain hyper-sensitivity between women with TTH or migraine and headache-free women?

With the aim of providing the most updated data, we considered any phase of the menstrual cycle (e.g., menstruation-bleeding, luteal phase, ovulation, or follicular phase) in women with regular or irregular cycles, in premenopausal women, and those with hormonal use.

### 2.2. Identify Relevant Studies

A systematic literature search was conducted with no data limitation to 31 December 2020 in the following electronic databases: PubMed, Medline, Web of Science, Scopus, and CINAHL. The database literature search was conducted by two different researchers (FCM, DPRdS). In case of disagreement, a third researcher (FAS) participated in the decision. The search strategy for TTH is shown in Table 1 and for migraine in Table 2.

### 2.3. Study Selection

This review used the PCC mnemonic (population, concept, and context) to define the inclusion criteria [25].

Population: Adult women (aged >18 years) diagnosed with TTH or migraine according to any edition of International Headache Society (IHS) criteria [26,27,28,29].

Concept: Comparison of pressure pain thresholds (PPT) between women with TTH or migraine and headache-free women.

Context: Observational studies including one or more groups with primary headache, such as TTH and/or migraine. Systematic reviews, narrative reviews, and meta-analyses or clinical trials were excluded.

Articles identified from the different databases were independently reviewed by two authors (FCM, DPRdS) with the assistance of the StArt program (copyright_version) designed by Federal University of Sao Carlos (Brazil). Authors were required to achieve a consensus on the included trials. In the case of discrepancy between the reviewers, a third author (CFdlP) decided the final inclusion or exclusion of the study.

### 2.4. Inclusion Criteria

(1) A group of women (age >18 years) diagnosed with TTH or migraine according to the IHS criteria. Studies including both women and men were also included but the main analysis, obviously, considered just the female group.
(2) A control group of healthy women without history of headache.
(3) Full text report published in Spanish or English as a journal article.
(4) Pressure pain sensitivity evaluated with PPTs assessed with a pressure algometer or dynamometer as the primary outcome.

### 2.5. Exclusion Criteria

(1) Studies assessing pain sensitivity with manual palpation or with other outcomes rather than an algometer (e.g., Von-Frey monofilament).
(2) Experimental-induced pain models (healthy subjects receiving a hypertonic saline injection or similar) of TTH or migraine.
(3) In those studies, evaluating different quantitative sensory tests, such as thermal or electrical pain thresholds, only PPTs measured with an algometer or dynamometer were included.

### 2.6. Data Charting Process

Data extraction was conducted using the Mendeley Desktop program. A data chart-ing form was developed for this scoping review to identify the variables that correspond with the research question. Data were extracted independently by two authors (FCM, DPRdS) using a data charting form, including title, authors, objectives, inclusion and exclusion criteria, size sample, size female sample, recruitment process, tool to assess PPT, average age of sample, and the use of any medication. Two researchers (FCM, DPRdS) completed the chart data and had to achieve a consensus on every item. In case of disagreement, a third researcher (FAS) participated in the decision to reach a resolution.

### 2.7. Methodological Quality

We used the Newcastle-Ottawa Scale, a star rating system, for evaluating the quality and the risk of bias of observational studies included in the review. The Newcastle-Ottawa Scale for case control studies consists of three items: selection (with 4 sections), comparability (1 section), and exposure (3 sections). Every section is formed by 2, 3, or 4 options and some of them are awarded a star if that criterion is clearly satisfied. The maximum score is nine stars. A score ≥7 stars means a study with low risk of bias [30].

## 3. Results

### 3.1. Study Selection

**Figure 1:**
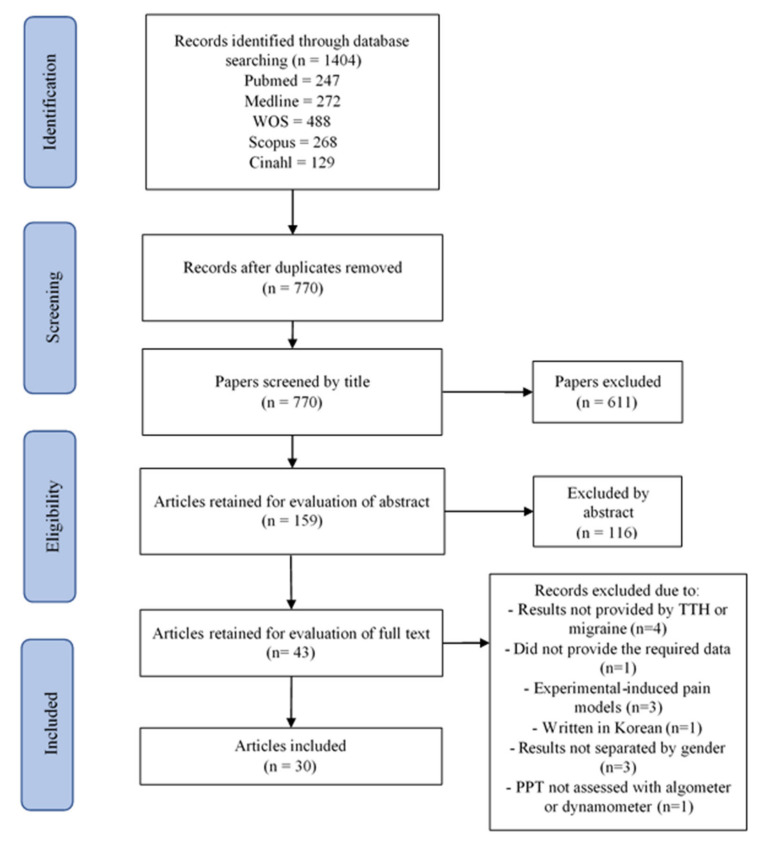
TTH process. PRISMA Extension for Scoping Reviews (PRISMA-ScR) flow diagram.

The initial search for TTH revealed a total of 1404 identified articles. After removing duplicates (*n* = 634), 770 were initially screened. A total of 611 studies were excluded after reading the title and another 116 after reading the abstract since they were not directly related to pressure pain sensitivity in TTH. After reading the full-text of the remaining 43 studies, the last 13 were excluded because: results were not provided by TTH or migraine [31,32,33,34], text did not provide the required data [35], they were experimental-induced pain models [36,37,38], it was written in Korean [39], results were not separated by gender [40,41,42], or PPT was not assessed with an algometer or dynamometer [43]. Finally, 30 studies satisfied all inclusion criteria and were included in the review of TTH (Figure 1) [6,7,8,9,10,11,44,45,46,47,48,49,50,51,52,53,54,55,56,57,58,59,60,61,62,63,64,65,66,67].

**Figure 2:**
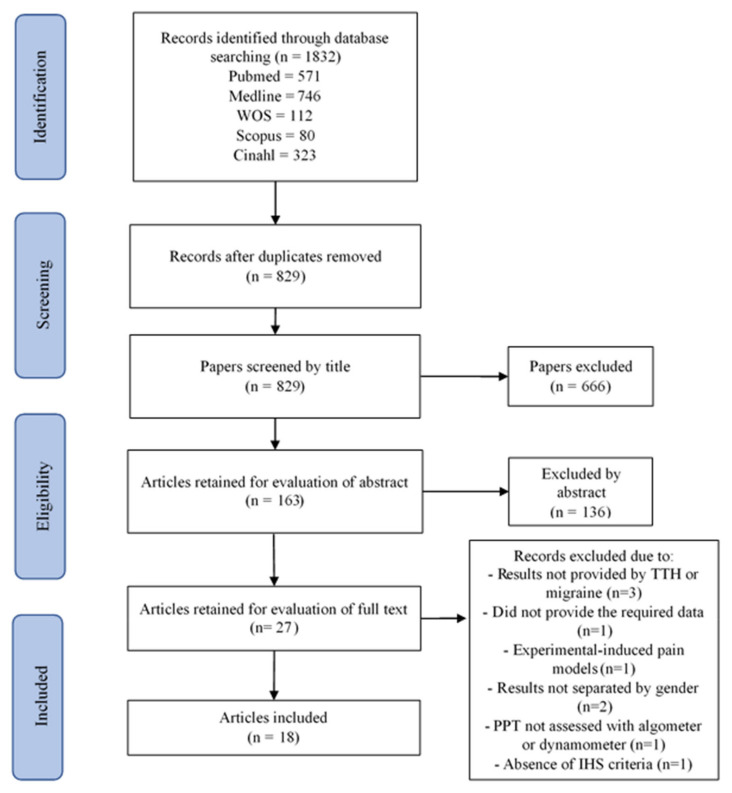
Migraine process. PRISMA Extension for Scoping Reviews (PRISMA-ScR) flow diagram.

The initial search for migraine revealed a total of 1832 identified articles. After removing duplicates (*n* = 1003), 829 were initially screened. A total of 666 studies were excluded after reading the title and another 136 after reading the abstract since they were not directly related to pressure pain sensitivity in migraine. After reading full-text of the remaining 27 studies, another nine were excluded because: results were not divided into TTH or migraine [31,32,34], text did not include the required data [68], it was an experimental-induced pain model [36], results were not separated by gender [69,70], PPT was not assessed with algometer or dynamometer [43], or due to the absence of IHS criteria [71]. Finally, 18 studies satisfied all inclusion criteria and were included in the review of migraine (Figure 2) [7,12,13,14,15,52,55,60,63,66,72,73,74,75,76,77,78,79].

### 3.2. Study Characteristics

Table 3 summarizes the characteristics of the studies investigating pressure pain sensitivity in women with TTH. The total sample of patients with TTH included in the studies was 1525 (986 women, 64.6%). A large number of women (≥50% of the sample) participated in the majority of the studies (*n* = 27, 93.34%) [6,8,9,10,11,23,44,45,46,47,48,50,52,53,54,55,56,57,58,59,60,61,62,63,64,65,66], except for just three where more males than females participated [48,51,67]. Six articles only recruited women [8,9,10,11,44,61]. The mean age of the sample was 38.8 years (SD 9.15, range from 18 to 76 years). Most of the selected studies used a pressure algometer, except for two studies using a dynamometer [9,62]. A headache diary [80] was used in fourteen articles to confirm the diagnosis of TTH or collect the clinical features of headache [10,11,15,44,45,47,48,49,50,51,53,54,64,65].

Patients with TTH were recruited from specific headache centres [64], random sampling from the general population [55,66], Danish Civil Registration [67], university centres or student population [9,44,45,46], advertisements in local media or newspapers [6,10,45,47,58,63], regular hospitals [9,11,48,49,50,51,56], outpatient clinics [52,53,54,57,59,65], tertiary care hospital [8], and university centres of ageing [63].

In nineteen (63.34%) studies, patients were asked for avoiding taking medication such analgesics or muscle relaxants 24–48 h before examination [7,9,10,11,44,45,46,47,48,49,50,51,52,53,58,59,60,61,63,64]. The remaining eleven (36.7%) did not take into account medication during the assessment procedure [6,8,54,55,56,57,62,63,65,66,67].

Table 4 summarizes the characteristics of the studies investigating pressure pain sensitivity in women with migraine. The total sample was 1000 patients with migraine (771 women, 77.1%). More women participated in the majority of these studies, except for one of them in which the same number of females and males participated [75]. Five articles only recruited women with migraine [12,13,14,15,73].

The average age of the sample with migraine was 39.7 with an SD of 8.77 years. Most of the selected studies used the pressure algometer, except for some of them reporting the use of dynamometer [14,73]. A diagnostic headache diary over two or four weeks [80] was not used in any study of migraine.

The recruitment process of the patients with migraine was done from tertiary hospitals [14,15,72], outpatient clinics [52,73], hospitals [75,77], pain clinics [79], advertisements at universities or local newspapers [63,74,78,79], university centres of ageing [63], random sample population [55,66], and social media such as Facebook [78,79].

Several studies excluded patients who had taken medication, such as analgesics or muscle relaxants, 24–48 h before examination [7,12,13,14,15,52,60,72,73,74,75,77,78,79], except for four in which this consideration was not taken into account [55,63,66,76]. Garrigós-Pedrón et al. [77] permitted the use of abortive pharmacological treatment during the assessment. 

### 3.3. Methodological Quality

**Figure 3:**
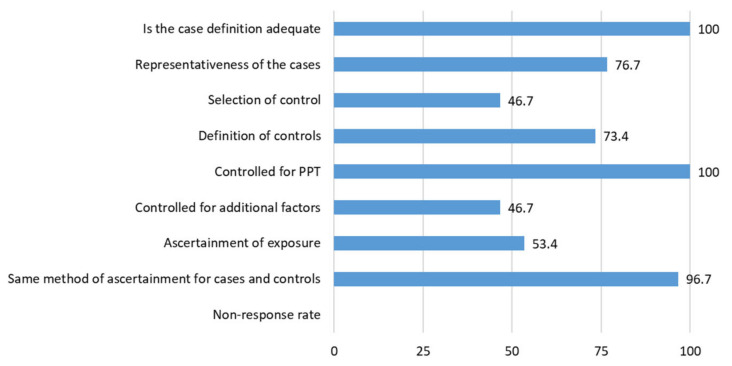
Percentage of assigned stars in tension-type headache (TTH) studies.

Table 5 shows the Newcastle-Ottawa Scale on each TTH study included in the review. A total of 19 (63.4%) studies showed a high risk of bias and the remaining 11 (36.7%) had aa low risk of bias according to the Newcastle-Ottawa Scale. The most common flaws were a failure to observe proper selection of controls and to control for other additional factors (Figure 3).

**Figure 4:**
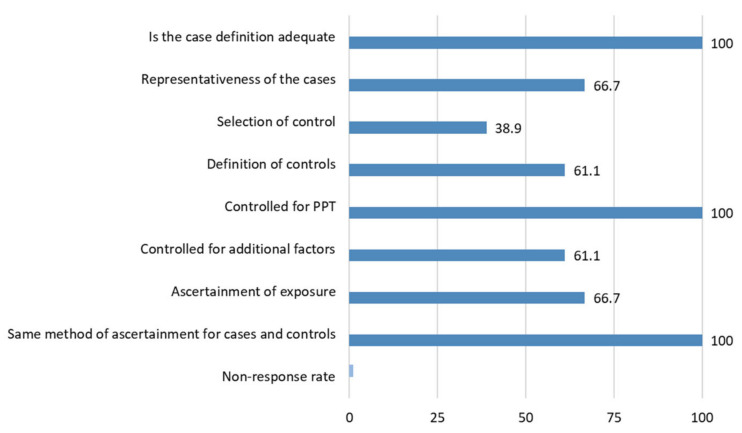
Percentage of assigned stars in migraine studies.

Table 6 shows the Newcastle-Ottawa Scale on each migraine study included in the review. A total of 11 (61.1%) studies showed a high risk of bias and the remaining seven (38.9%) had a low risk of bias according to the Newcastle-Ottawa Scale. The most common flaw was a failure to properly select controls (Figure 4).

### 3.4. Consideration of Menstrual Cycle

The days since the last menstruation were just recorded in one of the thirty studies (3.3%) investigating pressure pain sensitivity in TTH [47]. No other data of menstrual information were recorded in any other study. Importantly, the lack of consideration of the menstrual cycle was not considered as a limitation in any of the articles. Engstrom et al. collected the days since last menstruation in both women with and without TTH [47]. Twenty TTH patients (11 females) and 29 controls (15 females), comparable for age and sex, were included in this study. In the control group, the average number days since menstrual cycle was 16.7 (SD 9.2) days, whereas in the TTH group it was 11.3 (6.4) days. Menstrual data were not compared or associated with any other feature. Nothing else related with menstrual cycle was reported [47].

Similarly, the number of days since last menstruation were only recorded in one study including women with migraine, interestingly conducted by the same group [74]. In this case, two studies recognized that not recording the menstrual aspect was considered as a limitation [77,78]. In addition, Garridos-Pedrón et al. also considered as a limitation not reporting oral contraceptive use of the female participants [77]. Engstrom et al. [74] collected data about the number of days since last menstruation in controls (mean: 18.9 days, SD: 8.6 days), interictal migraine (mean: 14.2, SD: 7.6 days), preictal migraine (mean: 15.0, SD: 13.9 days), and postictal migraine (mean: 13.8, SD: 10.1 days) moments. Again, this menstrual data were not compared or associated with anything and no further data related with menstrual cycle were provided.

Interestingly, Strupf et al. included four patients (20% of their total sample size) with menstrual migraine [79], whereas Sales Pinto et al. included women with menstrual migraine associated with another primary headache [13]. Neither study considered the moment of the menstrual cycle in their assessments.

## 4. Discussion

### 4.1. Findings

This scoping review aimed to identify if the phase (menstruation-bleeding, luteal phase, ovulation, or follicular phase) of the menstrual cycle was considered as a cofounder factor in studies investigating pressure pain sensitivity between patients with TTH or migraine and healthy controls. The results of this review identified that the phase of the menstrual cycle has not been consistently considered in studies published to date investigating sensitivity to pressure pain in primary headaches, such as TTH or migraine, although evidence supports a potential relevance of the menstrual cycle in pain perception [17,18,81]. Only one study including individuals with TTH [47] and one including migraine patients [74] considered it relevant to include data about the last day since menstruation (but without specifying more data about this). However, these data were not compared or associated with anything else in these studies.

Similarly, an interesting finding was that just two studies including women with migraine considered the lack of menstrual information as a limitation [77,78]. Considering that more women are included in studies investigating primary headache, menstruation could be an important factor influencing pain sensitivity. We do not currently know if differences between women with migraine or TTH and headache-free women are due to “pain status” or influenced by a “menstrual status”. For instance, if headache patients are assessed in a follicular phase or other of the menstrual cycle, whereas control women are assessed during menstruation, ovulation, or a different phase of the menstrual cycle, between-group differences observed in PPTs can be related to the headache status (headache or control) but also to the menstrual cycle. In fact, this would be highly important in studies investigating pain sensitivity in women with chronic migraine, since menstrual-cycle disorders and dysmenorrhea are more prevalent in this population [82]. Future studies comparing women with migraine or TTH (no menstrual migraine) and healthy women should consider this and evaluate all participants, either patients or controls, in the same phase of the menstrual cycle and if differences between patients and controls are different depending on the phase of the menstrual cycle. This could be highly relevant since the menstrual cycle exhibits cyclic variations with an increased pain sensitivity during menstruation, suggesting that females could have lower PPTs during luteal and ovulation phases due to low levels of progesterone [81].

In fact, it has been already considered that females and males could have comparable detection thresholds for cold pain and ischemic pain while PPTs could be lower in females than males [83]. Similarly, Teepker et al. found that conditioned pain modulation inhibition neither differed between women with migraine and healthy women nor varied over the menstrual cycle [84].

### 4.2. Strengths and Limitations

The result from this scoping review should be extrapolated according to its strengths and limitations. The first strength was the use of different databases to avoid limiting the search and to include all articles fulfilling eligibility criteria. The second strength was the inclusion of studies without a limit date of publication. Third, we systematically evaluated all studies for determining the risk of bias and the inclusion of phase of menstrual cycle as a factor for being considered in PPT assessments. Among the limitations, although we included a total of 30 studies with TTH and 18 with migraine, almost 60% of the studies exhibited high risk of bias. Secondly, most studies were cross-sectional and no longitudinal studies investigating the time course of PPT have been conducted. Finally, the sample size of some of the studies was small, although this limitation does not restrict the extrapolation of our results in relation to the consideration of the phase of the menstrual cycle. In fact, the lack of information in relation to the investigated topic permits to determine different research lines for future studies.

### 4.3. Reliability and Validity of Pressure Pain Thresholds

Pressure pain threshold (PPT) is a static measure of pain reflecting the basal state of pain perception in relation to the pressure experienced a patient [85]. In fact, PPT is one of the quantitative sensory tests most commonly used for characterization of TTH [5] and migraine [4]. Nevertheless, its reliability and validity are controversial. Several studies reported good to excellent intra- and inter-rater reliability (intraclass correlation coefficient (ICC) > 0.70) when PPT are assessed on healthy subjects [86] or in individuals with different pain conditions [87,88]. Most studies calculate PPT as the mean of three consecutive trials assessed on the same point. However, evidence suggests that scores obtained at the first assessment are usually significantly higher than the two succeeding ones [89]. If this difference reaches 50kPa, it is recommended to conduct a fourth measurement and discard the highest value, although this is a recommendation not justified by evidence. More recent studies suggest that two measurements reduces the measurement error and presents excellent reliability (ICC ranging from 0.80 to 0.97) [90,91]. Most studies included in this scoping review conducted three measurements and calculated the means, but they did not calculate their internal reliability of PPT assessments. Therefore, results should be considered with caution. It would be recommended that futures studies comparing pressure sensitivity between women with headache and headache-free women calculate their own reliability data.

Another important topic is to determine if differences between headache patients and controls are clinically relevant and should be considered real. This is a topic of current debate since minimal detectable change (MDC) for PPT depends on the area of assessment and the population. For instance, Mailloux et al. 2021 [91] reported that the MDC for PPT ranged from 28.71 to 50.56 kPa in healthy subjects in the lumbar spine and the upper extremity. Walton et al. 2011 [87] found an MDC of 42.7 kPa for the cervical spine and of 86.3kPa for the tibialis anterior in asymptomatic subjects. In headache patients, Romero-Morales et al. [59] determined that a difference of 16.18kPa in the temporalis muscle and of 78.94kPa within the upper trapezius could be considered as real difference between people with TTH and headache-free subjects. Therefore, the comparison of pressure pain hyperalgesia between women with TTH or migraine against headache-free women should be accounted for according to these considerations.

This discussion increases in relevance when, in addition, considering the phase of the menstrual cycle, since some between-group variations could be related to the fact that two participating women are in a different phase of the cycle. Nevertheless, as will be discussed in the next section, studies determining the time course of pressure pain sensitivity throughout the different phases of the menstrual cycle are clearly needed. According to available data on MDC, the variations observed in PPTs between the different phases of the menstrual cycle should range between 50kPa and 100kPa depending on the area of assessment for determining real differences in sensitivity to pressure pain during the different phases of the menstrual cycle.

### 4.4. Future Research Directions

This review highlights the lack of information regarding consideration of the phase of the menstrual cycle in studies comparing pressure pain sensitivity between women with primary headaches, such as TTH or migraine, and healthy women and opens several questions for future research. First, further studies are needed to systematically determine if pain sensitivity is different throughout the different phases, e.g., menstruation-bleeding, luteal phase, ovulation, or follicular phase, of the menstrual cycle in both healthy women and women with headaches. In such a scenario, proper determination of the phase of the menstrual cycle when women are assessed will be highly important. For instance, determining the phase of the cycle by monitoring follicular development with ultrasound and measurement of estrogen/progestin blood measurements would be of high importance. With that information, future studies assessing pain sensitivity in women with migraine or TTH should evaluate all participants in the same phase of the menstrual cycle for avoiding an effect of this cofounder factor.

Similarly, medication intake of analgesics or muscle relaxants modifies pain perception as assessed with PPTs. In fact, a high proportion of studies included in this scoping review excluded patients who had taken analgesics or muscle relaxants 24–48 h before examination. However, regular consumption was not regularly controlled in most studies. This cofounder factor should be also considered in future studies. In such a scenario, another aspect to consider would be the use of hormonal contraception. Although the use of oral contraceptives is associated with an increased migraine intensity (at least at the end of menstruation), no effects on detection and pain thresholds has been observed in a small sample of migraineurs [81]. We believe that the differences in pain sensitivity in this group of women using any type of contraception may require separate studies.

Similarly, another subgroup of women to be considered is those with menstrual migraine. A review of studies applying neurophysiological procedures to test pain-related changes during the menstrual cycle in women with menstrual migraine found a fluctuation of the central modulation of pain across the menstrual phases, with a prevalence of excitatory versus inhibitory control in the premenstrual period [17]. Therefore, we do not know if the effect of menstrual cycle would be similar in this group of patients.

## 5. Conclusions

This scoping review found that observational studies examining sensitivity to pressure pain in women with TTH or migraine did not consistently consider the phase of the menstrual cycle, suggesting that the influence of menstrual cycle phase in observational studies on headache may likely be underestimated. Consequently, ignoring the effects of this cofounder factor may result in differences in PPTs between these primary headaches and healthy controls that could be related to the phase of the menstrual cycle and not just to the patient condition. Future studies investigating pressure pain sensitivity in TTH or migraine should consider the phase of the menstrual cycle in their evaluations.

## Figures and Tables

**Table 1:** Search Strategy for Tension-Type Headache Studies.

Pubmed
#1 “Tension-Type Headache” [Mesh] #2 “Tension-Type Headache” #3 “Tension Headache” #4 “Stress Headache” #5 “Psychogenic Headache” #6 “Idiopathic Headache” #7 “Tension Vascular Headache” #8 “Tension-Vascular Headache” #9 #1 OR #2 OR #3 OR #4 OR #5 OR #6 OR #7 OR #8 #10 “Hyperalgesia” [Mesh] #11 “Hyperalgesia” #12 “Sensitization” #13 “Pain sensitivity” #14 “Pressure pain threshold” #15 “Algometry” #16 “Pain threshold” #17 #10 OR #11 Or #12 OR #13 OR #14 OR #15 OR #16 #18 #9 AND #17 ((“Tension-Type Headache”[Mesh]) OR (“Tension-Type Headache”[Title/Abstract] OR “Tension Headache” [Title/Abstract] OR “Stress Headache”[Title/Abstract] OR “Psychogenic Headache”[Title/Abstract] OR “Idiopathic Headache”[Title/Abstract] OR “Tension Vascular Headache”[Title/Abstract] OR “Tension-Vascular Headache”[Title/Abstract])) AND ((“Hyperalgesia”[Mesh]) OR “Hyperalgesia”[Title/Abstract] OR “Sensitization”[Title/Abstract] OR “Pain sensitivity”[Title/Abstract] OR “Pressure pain threshold”[Title/Abstract] OR “Algometry”[Title/Abstract] OR “Pain threshold”[Title/Abstract])
**Filters: Title/Abstract + Mesh** **→ Results: 247**
**WOS**
#1 TS = (“hyperalgesia” OR “sensitization” OR “pain sensitivity” OR “pressure pain threshold” OR “algometry” OR “pain threshold”) #2 TS = (“Tension type headache” OR “tension-type headache” OR “idiopathic headache” OR “stress headache” OR “psychogenic headache” OR “tension headache” OR “tension vascular headache” OR “tension-vascular headache”)#3 #1 AND #2
**Filters: Web of Science Core Collection** **→ Results: 488**
**Scopus**
TITLE-ABS (“hyperalgesia” OR “sensitization” OR “pain sensitivity” OR “pressure pain threshold” OR “algometry” OR “pain threshold”) AND TITLE-ABS (“Tension type headache” OR “tension-type headache” OR “idiopathic headache” OR “stress headache” OR “psychogenic headache” OR “tension headache” OR “tension vascular headache” OR “tension-vascular headache”)
**Filters: TITLE-ABS** **→ Results: 268**
**Medline (via EBSCO)**
(“hyperalgesia” OR “sensitization” OR “pain sensitivity” OR “pressure pain threshold” OR “algometry” OR “pain threshold”) AND (“Tension type headache” OR “tension-type headache” OR “idiopathic headache” OR “stress headache” OR “psychogenic headache” OR “tension headache” OR “tension vascular headache” OR “tension-vascular headache”)
**Results: 269**
**Cinahl (via EBSCO)**
(“hyperalgesia” OR “sensitization” OR “pain sensitivity” OR “pressure pain threshold” OR “algometry” OR “pain threshold”) AND (“Tension type headache” OR “tension-type headache” OR “idiopathic headache” OR “stress headache” OR “psychogenic headache” OR “tension headache” OR “tension vascular headache” OR “tension-vascular headache”)
**Results: 129**

**Table 2:** Search Strategy for Migraine Studies.

Pubmed
#1 “Migraine Disorders” [Mesh] #2 “Migraine with Aura” [Mesh] #3 “Migraine without Aura” [Mesh] #4 “Ophthalmoplegic Migraine” [Mesh] #5 “Migraine” #6 (“Migraine Disorders” [Mesh] OR “Migraine with Aura” [Mesh] OR “Migraine without Aura” [Mesh] OR “Ophthalmoplegic Migraine”[Mesh]) AND (“hyperalgesia”[Title/Abstract] OR “sensitization”[Title/Abstract] OR “pain sensitivity” [Title/Abstract] OR “pressure pain threshold” [Title/Abstract] OR “algometry” [Title/Abstract] OR “pain threshold”[Title/Abstract])
**Filters: Title/Abstract + Mesh** **→ Results: 571**
**WOS**
#1 TS = (“hyperalgesia” OR “sensitization” OR “pain sensitivity” OR “pressure pain threshold” OR “algometry” OR “pain threshold”) #2 TS = (“Migraine Disorders” OR “Migraine with Aura” OR “Migraine without Aura” OR “Ophthalmoplegic Migraine”) #3 #1 AND #2
**Results: 112**
**Scopus**
TITLE-ABS (“hyperalgesia” OR “sensitization” OR “pain sensitivity” OR “pressure pain threshold” OR “algometry” OR “pain threshold”) AND TITLE-ABS (“Migraine Disorders” OR “Migraine with Aura” OR “Migraine without Aura” OR “Ophthalmoplegic Migraine”)
**Filters: TITLE-ABS → Results: 80**
**Medline (via EBSCO)**
(“hyperalgesia” OR “sensitization” OR “pain sensitivity” OR “pressure pain threshold” OR “algometry” OR “pain threshold”) AND (“Migraine Disorders” OR “Migraine with Aura” OR “Migraine without Aura” OR “Ophthalmoplegic Migraine”)
**Results: 740**
**Cinahl (via EBSCO)**
(“hyperalgesia” OR “sensitization” OR “pain sensitivity” OR “pressure pain threshold” OR “algometry” OR “pain threshold”) AND (“Migraine Disorders” OR “Migraine with Aura” OR “Migraine without Aura” OR “Ophthalmoplegic Migraine”)
**Results: 323**

**Table 3:** Characteristics of studies including women with tension-type headache (TTH).

Study	Objective (In Relation to Pressure Pain Sensitivity)	Inclusion Criteria	Exclusion Criteria	Tool to Assess PPT	Patients with TTH (F/M)	Mean Age
Ashina et al., 2005 [64]	To compare whether intramuscular and cutaneous pain sensitivity in cephalic region and in limb differs between patients with CTTH and healthy controls.	Patients with a diagnosis of CTTH according to the ICHD criteria, first edition (1988) and age between 18 and 65 years.	A history of more than one day with migraine per month; use of any kind of daily medication including prophy- lactic headache therapy but not oral contraceptives; excessive alcohol use; and serious somatic or psychiatric disorders.	Algometer Somedic	20 14/6	46
Bendtsen et al., 1996 [65]	To compare PPT and pressure pain tolerance thresholds between patients with CTTH and healthy controls	Patients with a diagnosis of CTTH according to ICHD criteria, first edition (1988). Patients with coexisting infrequent migraine (</= 1 day per month)	Patients suffering from serious somatic or psychiatric diseases and abusers of analgesics.	Electronic pressure algometer (Somedic)	40 25/15	40
Bovim, 1992 [7]	To compare PPT between patients with TTH, migraine, cervicogenic headache, and healthy controls.	Patients with a diagnosis of TTH according to ICHD criteria, first edition (1988).	NR	Algometer PTH-AF2, Pain Threshold Meter.	17 8/9	37
Buchgreitz et al., 2006 [66]	To evaluate pain perception in primary headaches by combining investigation of tenderness by manual palpation, PPT and SR-functions in 1300 persons from the general population in Denmark.	Patients with a diagnosis of episodic TTH according to the ICHD criteria, second edition (2004) in a large population living in Denmark.	Migraineurs with coexisting FETTH or CTTH.	Algometer Somedic	108 70/38	NR
Buchgreitz et al., 2008 [67]	To explore the cause-effect relationship between increased pain sensitivity (decreased PPT) and the development of headache between FETTH, CTTH, migraine and healthy controls.	Patients with a diagnosis of episodic TTH according to the ICHD criteria, second edition (2004). All subjects who were still living in Denmark in 2001 and who were capable of answering written and verbal questions.	Migraineurs with coexisting FETTH or coexisting CTTH and subjects with coexisting migraine with FETTH and CTTH.	Algometer (Somedic)	388 190/198	56
Caamaño-Barrios et al., 2019 [44]	To compare PPT over symptomatic and distant pain-free nerve trunk areas between women with TTH and healthy controls	Consecutive women with a diagnosis of TTH according to the ICHD criteria, third edition (2013)	Chronic headaches; other primary/secondary headache including medication overuse headache; head/neck trauma (i.e., whiplash); cervical herniated disk or cervical osteoarthritis (medical records); any systemic degenerative disease; diagnosis of fibromyalgia; had received anesthetic blocks or any physical treatment in the previous six months; or pregnancy.	Algometer (Somedic)	32 32/0	22
Cathcart et al., 2008 [45]	To examine interactions between daily stress, pain sensitivity, and headache activity in CTTH sufferers.	Patients with a diagnosis of CTTH according to the ICHD criteria, first edition (1988)	NR	Mechanical algometer	16 8/8	34
De Cássia Correia Kälberer Pires et al., 2017 [9]	To estimate differences in PPT in cranio-cervical muscle TrPs between women with unilateral migraine or TTH, compared to asymptomatic women.	Women suffering from unilateral migraine, FETTH or CTTH lasting over a year according to the ICHD criteria, second edition (2004).	Women with a previous history of neck trauma or whiplash, and those with other primary headaches and a history of headache lasting less than a year.	Dynamometer	20 20/0	34
Drummond et al., 2011 [46]	To determine whether the inhibitory effect of acute limb pain on pain to mechanical stimulation of the forehead is compromised in individuals with FETTH.	Individuals who reported at least 1 episode of headache per month for FETTH according to the ICHD criteria, second edition (2004)	Individuals with a history of migraine and those who took prescribed medication to treat their headaches or any other medical condition.	Algometer	34 23/11	22
Engstrøm et al., 2014 [47]	To evaluate the relationship between sleep quality and pain thresholds (PPT) in healthy controls and TTH patients.	Subjects with ETTH or CTTH according to the ICHD criteria, second edition (2004).	Subjects with known sleep disorders, coexisting frequent migraine, other major health problems or pregnancy, moderate or severe sleep apnoea defined as apnoea hypopnoea index (AHI).	Algometer (Somedic)	20 11/9	41
Fernández-de-las-Peñas et al., 2009 [11]	To investigate mechanical pain sensitivity distribution (PPT) over the trapezius muscle in patients with CTTH, strictly unilateral migraine and healthy controls.	Patients with a diagnosis of CTTH according to the ICHD criteria, second edition (2004).	Participants with previous whiplash or neck trauma and other primary headaches,	Algometer (Somedic)	20 20/0	39
Fernández-de-las-Peñas et al., 2008 [48]	To evaluate differences in PPT levels for the first division of the trigeminal nerve (V1) between patients with CTTH and controls; and	Patients with a diagnosis of CTTH according to the ICHD criteria, second edition (2004).	None of the patients fulfilled the criteria for other primary headaches, and there was no indication for secondary headaches based on history, physical, and neurological examinations.	Mechanical pressure algometer	20 12/8	35
Fernández-de-las-Peñas et al., 2007 [49]	To compare PPT in both cephalic and neck points between CTTH patients and healthy participants	Patients presenting with CTTH associated with pericranial tenderness according to the ICHD criteria, second edition (2004).	Medication-overuse headache as defined by the IHS was ruled out in all cases	Pressure Threshold Meter, algometer	25 12/13	41
Fernández-de-las-Peñas et al., 2007 [50]	To analyze if the decrease in PPT was related to the presence of TrPs in the temporalis muscle in patients with CTTH and healthy controls.	Subjects with CTTH according to the ICHD criteria, second edition (2004).	Patients with mixed headache.	Mechanical pressure algometer	30 21/9	39
Fernández-de-las-Peñas et al., 2007 [51]	To analyse if the decrease in PPT in the upper trapezius muscle was related to the presence of TrPs in the upper trapezius muscle in patients with CTTH and healthy subjects.	Subjects with CTTH according to the ICHD criteria, second edition (2004).	Medication-overuse headache as defined by the IHS was ruled out in all cases	Mechanical pressure algometer	20 9/11	36
Fernández-de-las-Peñas et al., 2008 [10]	To characterize hypersensitivity (decreased PPT) of the temporalis muscle in CTTH patients and healthy controls	Women with CTTH according to the ICHD criteria, second edition (2004).	Other primary headaches	Electronic pressure algometer (Somedic)	15 15/0	40
Filatova et al, 2008 [52]	To investigate central sensitization in chronic headache with a variety of methods and compare this phenomenon across CM and CTTH.	Patients with IHS-defined CM, CTTH or mixed chronic headache, according to the ICHD criteria, second edition (2004)	Age under 18 or over 65, the presence of peripheral neuropathy, dermato- logical disease, chronic pain in another location, major psychiatric disorder.	Hand-held pressure algometer	25 23/2	39
Jensen, 1995 [53]	To analyze the relative importance of central and peripheral nociceptive factors by assessing PPT and tolerance thresholds	Patients with TTH during at least 1 year, according to the ICHD criteria, first edition (1988), and age between 18 and 70 years.	Daily headache, migraine more than 1 day/month, cluster headache or trigeminal neuralgia, other neurological, somatic or psychiatric disorders, concurrent ingestion of major medications including migraine prophylactics, any form of drug abuse or dependency including large amounts of plain analgesics.	Electronic pressure algometer (Somedic)	28 17/11	45
Jensen et al., 1998 [54]	To compare the mechanical and the thermal pain sensitivity in TTH with and without disorders of pericranial muscles.	Patients with TTH according to the ICHD criteria, first edition (1988). Duration of TTH for at least 1 year and age between 18 and 70 years.	Migraine more than 1 day per month; cluster headache; trigeminal neuralgia; other neurological, systemic, or psychiatric disorders; ingestion of major medications including prophylactics for migraine or other headaches; or any form of drug abuse or dependency such as daily ergotamine or large amounts of plain analgesics.	Electronic pressure algometer (Somedic)	58 36/22	41
Jensen et al., 1993 [55]	To evaluate the possible role of pericranial myofascial nociception (PPT) in headache pathogenesis.	Patients with CTTH diagnosis according to the ICHD criteria, first edition (1988).	Comorbid migraine attacks more than 30 days in the previous year	Pressure algometry	158 96/62	NR
Langemark et al., 1989 [56]	To compere the nociceptive thresholds of mechanical and thermal stimuli in patients with CTTH.	A history of TTH according to the ICHD criteria, first edition (1988) of at least 6 months duration and no more than 14 headache-free days/month.	Patients with a history of FM (more than one attack per month)	Pressure algometer	32 22/10	40
Malo-Urriés et al., 2020 [6]	To evaluate and compare sensory function in the trigeminocervical region in patients with CH, MH, and TTH and healthy controls.	Patients with headache according to the ICHD criteria, third edition (2013) for CH, MH, and TTH, respectively.	Other type of headache	Pressure algometer (Somedic)	71 59/12	38
Mazzotta et al., 1997 [57]	To confirm if chronic headache and migraine patients have a defect in the antinociceptive system by assessing PPT.	Patients with a diagnosis of ETTH according to the ICHD criteria, first edition (1988).	NR	Pressure algometer (Somedic)	30 20/10	36
Palacios Ceña et al., 2016 [8]	To compare differences in widespread PPT between women with FEETH, CTTH and healthy controls.	Patients with a diagnosis of FETTH or CTTH according to the ICHD criteria, third edition (2013).	(1) other primary/secondary headache; (2) medication overuse headache as defined by the ICHD-III; (3) cervical or head trauma; (4) pregnancy; (5) history of cervical herniated disk or cervical osteoarthritis; (6) any systemic degenerative disease, eg, rheumatoid arthritis, lupus erythematous; (7) comorbid diagnosis of fibromyalgia syndrome; (8) receiving anesthetic block within the previous 6 months; or (9) receiving physical treatment previous 6 months.	Electronic pressure algometer (Somedic)	92 92/0	47-48
Peddireddy et al., 2009 [58]	To investigate whether jaw-stretch reflex and PPT of pericranial muscles in patients with CTTH differed from that of healthy individuals and between males and females	Subjects who had experienced headache > 16 days/month and with CTTH diagnosis according to the ICHD criteria, second edition (2004)	NR	Handheld electronic algometer (Somedic)	30 15/15	45
Romero-Morales et al., 2017 [59]	To evaluate the MCDs in the PPTs of the temporalis and upper trapezius muscles in patients with and without TTH.	Individuals with TTH according to the ICHD criteria, second edition (2004), guidelines for TTH.	Diagnosis of migraine (>1 episode/month), diabetes, or fibromyalgia; surgery in the upper-limb or cervical regions; secondary headache; depression; neurologic or cardiovascular disease; temporomandibular disorders; pregnancy; or physical therapy treatment in the previous 6 months. Patients who were taking prophylactic or analgesic medication.	Mechanical algometer (FDK/FDN)	60 32/28	36
Sandrini et al., 1994 [60]	To address some of the current methodological limitations by comparing the results obtained with three different procedures in TTH, MH, and controls.	Subjects with CTTH or MH without aura patients (attacks frequency/range, 1-3/month), who were diagnosed according to the ICHD criteria, first edition (1988). Patients with an illness duration of more than 5 years.	NR	Electronic pressure algometer (Somedic)	44 25/19	32
Schoenen et al., 1991 [61]	To determine PPT in pericranial muscles as well as at an extracephalic site, the Achilles tendon, in patients with CTTH, migraineurs or healthy controls.	Females presenting with CTTH according to the ICHD criteria, first edition (1988).	Patients taking more than 3 analgesic tablets/week or treated with psychotropic drugs or patients who had taken such drugs less than 24 h before investigation.	Pressure algometer (Somedic)	32 32/0	41
Stroppa-Marques et al., 2017 [62]	To analyze the PPT of the SCM, SO, and UT muscles in individuals with ETTH.	Adults of both genders, ranging from 18 to 27 years of age, with ETTH according to the ICHD criteria, second edition (2004).	Individuals with neurological or systemic diseases, previously diagnosed psychiatric disorders, cachexia, postural changes in treatment, lesions in the upper limb cingulum, diagnosis of CTTH or any other type of headache	Pressure dynamometer (algometer) (Kratos)	30 21/9	20
Uthaikhup et al., 2009 [63]	To investigate whether decreased PPT (hypersensitivity) were present in elders with different CTTH compared with elders without headache.	Patients diagnosed according to the ICHD criteria, second edition (2004).	Comorbid medical conditions that might interfere with pain measures. Patients with headache if they reported 2 or more types of headache or had headaches which had been diagnosed medically as secondary or associated with neurologic or systemic disorders.	Electronic algometer (Somedic)	10 6/4	65

NR: No reported, TMDSQ: Temporomandibular disorder screening questionnaire, PPT: Pressure pain threshold, PT: Pain threshold, HIS: International Headache Society, HIT-6: Headache Impact Test” 6, F: Female, M: Male, CTTH: Chronic Tension Type-Headache, TrPs: Trigger Points, ETTH: Episodic Tension Type-Headache, FM: Frequent migraine, CM: Chronic migraine, CH: Cluster headache, MH: Migraine Headache, SCM: Sternocleidomastoid, SO: Suboccipital, UT: upper trapezius, QST: Quantitative Sensory Tests.

**Table 4:** Characteristics of studies including women with migraine.

Study	Objective (In Relation to Pressure Pain Sensitivity)	Inclusion Criteria	Exclusion Criteria	Tool to Assess PPT	Patients with MH (F/M)	Mean Age
Barón et al., 2016 [72]	To compare topographical PPT over the scalp in patients with MH, grouping them on episodic/chronic or unilateral/bilateral symptoms.	Patients with migraine diagnosed according to the ICHD criteria, third edition (2013)	(1) other primary or secondary headaches, including medication overuse headache accordingly to the ICHD3 criteria; (2) history of neck or head trauma (i.e., whiplash); (3) pregnancy; (4) systemic disease, e.g., rheumatoid arthritis, lupus erythematous; (5) diagnosis of fibromyalgia; (6) previous treatment with botulinum toxin; or (7) anesthetic block within the past 3 months.	Mechanical algometer	162 100/62	38-39
Bevilaqua Grossi et al., 2011 [73]	To evaluate the cranio-cervical PPT values in women with EM and CM, relative to controls.	Women from 20 to 60 years with EM without aura or with CM, diagnosed according to the ICHD criteria, second edition (2004).	Other primary headaches, use of analgesic medication over the 24 h before the evaluation with pressure algometry medication overuse headache, women who reached a PPT value above the maximum permitted by the apparatus (20 kg) during calibration (palpation of the thenar region) and women diagnosed with neuropathic pain.	Digital manual dynamometer (DDK-10, Kratos)	29 29/0	37
Bovim et al., 1992 [7]	To compare PPT measurements between cervicogenic headache, migraine without aura, and TTH.	A diagnosis of TTH according to the ICHD criteria, first edition (1988).	NR	Algometer, PTH-AF2, Pain Threshold Meter.	26 20/6	36
Buchgreitz et al., 2006 [66]	To evaluate pain perception in primary headaches by combining investigation of tenderness by manual palpation, PPT and SR-functions in 1300 persons from the general population in Denmark.	A diagnosis of migraine according to the ICHD criteria, second edition (2004). To live in Denmark.	Migraineurs with coexisting FETTH or CTTH.	Algometer	60 42/18	NR
Engstrøm et al., 2013 [74]	To compare subjective and objective sleep quality and arousal in migraine and to evaluate the relationship between sleep quality and pain thresholds (PT) in controls, interictal, preictal and postictal migraine.	Subjects with two to six episodes per month of migraine (M), with- (MA) and without aura (MwoA), diagnosed according to the ICHD criteria, second edition (2004).	Subjects with coexisting FM and TTH, other major health problems (sleep disease, hypertension, infection, neoplastic disease, neurological disease, CNS-implants, cardial or pulmonary disease), chronic or acute pain, regular use of neuroleptic, antiepileptic or antidepressant drugs, analgesics, or drugs for migraine prophylaxis the last four weeks), or subjects who were pregnant.	Algometer	84 58/26	38
Fernández-de-las-Peñas et al., 2009 [75]	To investigate differences in PPT in nerve trunks between patients with strictly unilateral migraine and healthy control participants;	A diagnosis of migraine according to the ICHD criteria, second edition (2004).	Medication-overuse headache	Mechanical pressure algometer	20 10/10	36
Fernández-de-las-Peñas et al., 2008 [76]	To analyse the differences in PPT and pericranial tenderness between patients with strictly unilateral migraine and healthy controls;	A diagnosis of migraine according to the ICHD criteria, second edition (2004).	NR	Pressure algometer	25 17/8	32
Fernández-de-las-Peñas et al., 2009 [12]	To calculate topographical pressure pain sensitivity maps of the temporalis muscle in a blind design in patients with strictly unilateral migraine compared with controls.	Patients presenting the following features typical of migraine according to the ICHD criteria, second edition (2004)	Other primary headaches	Pressure algometer	15 15/0	36
Filatova et al., 2008 [52]	To investigate central sensitization in chronic headache comparing CM and CTTH.	Patients with IHS-defined CM, CTTH or mixed chronic headache according to the ICHD criteria, second edition (2004).	Age under 18 or over 65, the presence of peripheral neuropathy, dermatological disease, chronic pain in another location, major psychiatric disorder.	Hand-held pressure algometer	25 23/2	44
Florencio et al., 2015 [14]	To investigate differences in PPT in the neck musculature between migraine patients and controls subjects.	Migraine patients diagnosed according to the ICHD criteria, third edition (2013).	Subjects with other primary headaches; medication overuse head- ache; pregnancy; systemic degenerative diseases such as rheumatoid arthritis, lupus erythematous, or other medical diseases affecting sensitivity, for example, such fibromyalgia or previous neck trauma (whiplash).	Digital manual dynamometer (DDK-10 Kratos)	30 30/0	37
Garrigós-Pedrón et al., 2019 [77]	To assess mechanical hyperalgesia in the trigeminal and extra-trigeminal region in patients with CM and TMD and to compare with a control group.	Diagnosis of CM according to the ICHD criteria, third edition (2013), and diagnosis of myofascial TMD, as defined by the Research Diagnostic Criteria for TMD (RDC/TMD).	Migraine crisis at the time of assessment, presence of other headache, another type of TMD, history of another chronic disease, history of neurological disease and/or dental problems and previous surgery or trauma to the upper body.	Analog algometer	52 48/4	46
Jensen et al., 1993 [55]	To evaluate the possible role of pericranial myofascial nociception in headache pathogenesis.	A migraine diagnosis according to the ICHD criteria, first edition (1988).	Migraineurs with concurrent TTH more than 30 days in the previous year	Pressure algometry	158 96/62	NR
Palacios Ceña et al., 2016 [15]	To investigate widespread PPT women with EM, CM and healthy controls.	Women with migraine diagnosed according to the ICHD criteria, third edition (2013).	1, other primary or secondary headache, including medication overuse headache; 2, history of neck/ head trauma; 3, pregnancy; 4, cervical herniated disk or cervical osteoarthritis on medical records; 5, any systemic medical disease; 6, comorbid diagnosis of fibromyalgia; or, 7, anesthetic block in the past 3 months.	Electronic pressure algometer	103 103/0	40-41
Sales Pinto et al., 2013 [13]	To evaluate the influence of the concomitant presence of myofascial pain on the PPT values of masticatory muscles in women with a migraine.	Women, with ages ranging from 18 to 60 years, diagnosed with EM, according to the IHS criteria (NR).	Patients with only menstruation-related migraine, chronic migraine, other primary headaches, secondary headaches, or systemic conditions (eg, fibromyalgia)	Digital algometer (KRATOS)	101 101/0	NR
Sandrini et al., 1994 [60]	To address methodological limitations by comparing the results obtained from TTH, MH, and controls.	Subjects with CTH or MH without aura patients diagnosed according to the ICHD criteria, first edition (1988). Patients with an illness duration of more than 5 years.	NR	Electronic pressure algometer	44 25/19	32
Scholten-Peeters et al., 2020 [78]	(1) To compare PPT during the preictal, ictal, postictal and interictal phases in people with migraine in both cephalic and extra-cephalic regions, and (2) To assess differences in mechanical sensitivity between people with migraine and healthy participants in both cephalic and extra- cephalic regions	People with migraine according to the ICHD criteria, third edition (2013), aged between 18 and 65 years and Dutch or English speaking.	Other types of headaches such as medication overuse headache, head or neck complaints within 2 months prior to the measurements, musculoskeletal painful conditions, psychiatric conditions, malignancy or other neuropathic pain states. Participants who received treatment for headache 48 h before the measurements or those who received botulinum toxin injections	Pressure algometer	19 16/3	47
Strupf et al., 2018 [79]	To find out if there are changes in pain thresholds and habituation between these groups and in a temporal association with migraine attacks and CTTH fluctuations.	Patients diagnosed with migraine with or without aura and CTTH according to the ICHD criteria, third edition (2013), with at least 15 headache days per month took part in the study.	NR	Electronic pressure algometer	21 20/1	30
Uthaikhup et al., 2009 [63]	To investigate whetherdecreased PTP (hypersensitivity) were different CTTH compared with elders without headache.	Patients diagnosed according to the ICHD criteria, second edition (2004) and the Cervicogenic Headache International Study Group’s criteria for cervicogenic headache.	Comorbid medical conditions that might interfere with pain measures (inflammatory arthritis, fibromyalgia, neurologic symptoms, cognitive disturbance, or psychiatric disorders). Patients with headache if they reported 2 or more types of headache or had headaches which had been diagnosed medically as secondary or associated with neurologic or systemic disorders.	Electronic algometer	26 18/8	66

NR: No reported, PPT: Pressure pain threshold, PT: Pain threshold, IHS: International Headache Society, F: Female, M: Male, FM: Frequent migraine, EM: Episodic migraine, CM: Chronic migraine, TMD: Temporomandibular Disorders, ICHD: International Classification of Headache Disorders.

**Table 5:** Risk of bias (the Newcastle-Ottawa Scale) of the included tension-type headache (TTH) studies.

		Ashina et al., 2005 [64]	Bendtsen et al., 1996 [65]	Bovim, 1992 [7]	Buchgreitz et al., 2006 [66]	Buchgreitz et al., 2008 [67]	Caamaño-Barrios et al., 2019 [44]	Cathcart et al., 2008 [45]	De Cássia Correia Kälberer Pires et al., 2017 [9]	Drummond et al., 2011 [46]	Engstrøm et al., 2014 [47]	Fernández-de-las-Peñas et al., 2007 [49]	Fernández-de-las-Peñas et al., 2007 [50]	Fernández-de-las-Peñas et al., 2007 [51]	Fernández-de-las-Peñas et al., 2008 [10]	Fernández-de-las-Peñas et al., 2008 [48]	Fernández-de-las-Peñas et al., 2009 [11]	Filatova et al., 2008 [52]	Jensen et al., 1993 [55]	Jensen, 1995 [53]	Jensen et al., 1998 [54]	Langemark et al., 1989 [56]	Malo-Urriés et al., 2020 [6]	Mazzotta et al., 1997 [57]	Palacios Ceña et al., 2016 [8]	Peddireddy et al., 2009 [58]	Romero-Morales et al., 2017 [59]	Sandrini et al., 1994 [60]	Schoenen et al., 1991 [61]	Stroppa-Marques et al., 2017 [62]	Uthaikhup et al., 2009 [63]	
S	1	★	★	★	★	★	★	★	★	★	★	★	★	★	★	★	★	★	★	★	★	★	★	★	★	★	★	★	★	★	★	1
	2	★	★		★	★	★	★	★	★		★	★	★	★	★	★		★	★	★	★	★		★	★	★				★	2
	3				★	★		★		★	★				★	★			★			★	★	★			★		★	★	★	3
	4	★	★		★	★	★	★	★	★			★	★	★	★	★		★				★	★	★	★	★	★		★	★	4
C	5	★	★	★	★	★	★	★	★	★	★	★	★	★	★	★	★	★	★	★	★	★	★	★	★	★	★	★	★	★	★	5
	6	★		★				★	★		★	★	★	★				★		★	★	★	★								★	6
E	7	★		★			★			★	★	★	★	★	★	★	★		★		★		★		★	★						7
	8	★	★	★	★	★	★	★	★	★	★	★	★	★	★	★	★	★	★	★	★	★	★	★	★	★		★	★	★	★	8
	9																															9
SC		L	H	H	H	H	H	L	H	L	H	H	L	L	L	L	L	H	L	H	H	H	L	H	H	H	H	H	H	H	L	

S: Selection, C: Comparability, E: Exposure, SC: Score, H: high risk of bias, L: low risk of bias, 1: Is the case definition adequate, 2: Representativeness of the cases, 3: Selection of control, 4: Definition of controls, 5: Controlled for PPT, 6: Controlled for additional factors, 7: Ascertainment of exposure, 8: Same method of ascertainment for cases and controls, 9: Non-response rate. A score ≥7 stars means a study with low risk of bias (L).

**Table 6:** Risk of bias (the Newcastle-Ottawa Scale) of the included studies in including women with migraine.

		Barón et al., 2016 [72]	Bevilaqua Grossi et al., 2011 [73]	Bovim, 1992 [7]	Buchgreitz et al., 2006 [66]	Engstrøm et al., 2013 [74]	Fernández-de-las-Peñas et al., 2007 [76]	Fernández-de-las-Peñas et al., 2009 [12]	Fernández-de-las-Peñas et al., 2009 [75]	Filatova et al., 2008 [52]	Florencio et al., 2015 [14]	Garrigós-Pedrón et al., 2019 [77]	Jensen et al., 1993 [55]	Palacios Ceña et al., 2016 [15]	Sales Pinto et al., 2013 [13]	Sandrini et al., 1994 [60]	Scholten-Peeters et al., 2020 [78]	Strupf et al., 2018 [79]	Uthaikhup et al., 2009 [63]	
S	1	★	★	★	★	★	★	★	★	★	★	★	★	★	★	★	★	★	★	1
	2	★	★		★		★	★	★		★	★	★	★			★		★	2
	3				★	★					★		★				★	★	★	3
	4	★			★			★			★	★	★	★		★	★	★	★	4
C	5	★	★	★	★	★	★	★	★	★	★	★	★	★	★	★	★	★	★	5
	6	★		★		★	★	★		★		★			★		★	★	★	6
E	7	★	★	★		★	★	★	★		★	★	★	★			★			7
	8	★	★	★	★	★	★	★	★	★	★	★	★	★	★	★	★	★	★	8
	9																			9
SC		L	H	H	H	H	H	L	H	H	L	L	L	H	H	H	L	H	L	SC

S: Selection, C: Comparability, E: Exposure, SC: Score, H: high risk of bias, L: low risk of bias, 1: Is the case definition adequate, 2: Representativeness of the cases, 3: Selection of control, 4: Definition of controls, 5: Controlled for PPT, 6: Controlled for additional factors, 7: Ascertainment of exposure, 8: Same method of ascertainment for cases and controls, 9: Non-response rate. A score ≥7 stars means a study with low risk of bias (L).

## Data Availability

The data presented in this study are available on request from the corresponding author.
